# Editorial: Metabolite-induced metabolic reprogramming in the immune system

**DOI:** 10.3389/fimmu.2026.1927032

**Published:** 2026-07-20

**Authors:** Jacqueline Oliva-Ramírez, Heriberto Prado-Garcia, Francisco Javier Sánchez-García

**Affiliations:** 1 Translational Molecular Pathology, MD Anderson Cancer Center, Houston, TX, United States; 2 Laboratorio de Onco-Inmunobiologia, Departamento de Enfermedades Crónico-Degenerativas, Instituto Nacional de Enfermedades Respiratorias, “Ismael Cosio Villegas”, Mexico City, Mexico; 3 Laboratorio de Inmunorregulación, Departamento de Inmunología, Escuela Nacional de Ciencias Biológicas, Instituto Politécnico, Nacional, Mexico City, Mexico

**Keywords:** immunity, immunometabolism, metabolic reprogramming, metabolite intervention, metabolite signaling

## The concept

Historically, the concept of metabolic reprogramming might be traced back to the 1920s, when Otto Warburg discovered that tumor cells, in opposition to non-transformed cells, obtain their energy primarily from glycolysis, even in the presence of oxygen (aerobic glycolysis). Later, the discovery that T lymphocyte co-stimulation is also a metabolic co-stimulation (1) prompted a wealth of research aimed at identifying the metabolic pathways preferentially used by immune cells under specific conditions of activation and differentiation. In due course, the field of Immunometabolism flourished and, along this, the finding that metabolites may function as signaling molecules (2). Lactate, a metabolite long-regarded as a “by-product” or “waste” of glycolysis, is now recognized as a pleiotropic cell-signaling molecule with profound implications in infection and cancer (3, 4); the signaling properties of itaconate, succinate, and fumarate (5), as well as of some microbiota-derived metabolites, such as acetate, butyrate, and propionate (6) are widely recognized. Nevertheless, when studying metabolic reprogramming of immune cells, immunologists have mostly used Pattern Recognition Receptors-ligands (LPS, β-glycan, etc.), whole microorganisms (BCG, *C. albicans*)*,* antigenic mixtures (cell-free extracts, etc.), TCR complex-stimulating reagents (anti-CD3 mAbs, etc.), as stimuli. Why not metabolites as inducers of metabolic reprogramming? Perhaps metabolite-induced metabolic reprogramming in the immune system, is a potentially rich path for inquiry (7).

The topic here presented comprises five papers that cover several aspects of macrophage metabolic reprogramming, metabolic regulation by transcriptional regulators of MHC class I and class II genes, regulation of neutrophil metabolism by platelets, microbial metabolites regulating immune cells and, the role of free heme on sepsis.

Wang et al. review how a gut-derived metabolite, trimethylamine N-oxide (TMAO), favors the development of several chronic inflammatory diseases, in particular inflammatory bowel disease (IBD) in which dysbiosis and microbiota-derived metabolites contribute to its pathogenesis. Trimethylamine is synthesized from choline or carnitine by *Firmicutes* and *Actinobacteria* present in the gut microbiota; Trimethylamine is then oxidized to TMAO in the liver. Paradoxically, although *Firmicutes* is decreased in the gut microbiota of IBD patients, TMAO levels are increased in these patients. Thus, other dietary components and altered microbiota might be the source of TMAO. TMAO activates the NLRP3 inflammasome *in vitro*, suggesting a crucial role in the pathogenesis of IBD. Furthermore, TMAO promotes inflammation by skewing macrophages towards a predominant M1 profile; other immune cells, such as dendritic cells and Th1 cells, are also stimulated by TMAO. Wang et al. propose that TMAO might be a therapeutic target for the treatment of IBD by means of dietary interventions.

Cui et al. review how metabolic reprogramming modulates macrophage phenotype and function across several pathologies, pointing out the important role of cell location in favoring specific metabolic profiles in macrophages and the maintenance of homeostasis. For instance, alveoli macrophages rely on lipid metabolism and to a lesser extent on glycolysis, whereas intestinal macrophages which are modulated by the diet and gut microbiota, mostly rely on fatty acid oxidation or oxidative phosphorylation. In chronic infections, pathogens reprogram immune cells metabolism either by the action of their own metabolites or by rewiring macrophage metabolic pathways i.e., pathogens modulate tissue resident macrophages and other immune cells present in the microenvironment. Cui et al. propose that understanding the specific metabolism of tissue-resident macrophages and identifying metabolic regulators might have valuable therapeutic potential for the treatment of infectious diseases.

Mosso-Pani et al. review on the interaction of platelets and neutrophils and point out that although platelets are known to maintain hemostasis and endothelial integrity, they are also important for neutrophil metabolic reprogramming.

The fact that neutrophil metabolism can be reprogrammed somehow contradicts the longstanding notion that neutrophils are primarily glycolytic, given their short lifespan and rapid response to pathogens. The metabolic processes taken place in the neutrophils mitochondria modulate neutrophils’ functions; for instance, neutrophil mitochondrial ROS production is favored by some bacterial products, such as N-formyl peptides.

Neutrophils and platelets may form neutrophil-platelet aggregates, which in turn engage in bi-directional modulation. Mosso-Pani et al. suggest that platelets, by means of platelet-derived growth factor, induce mitochondrial fission in neutrophils to increase the oxidative burst and neutrophil extracellular traps formation. This hypothesis awaits confirmation.

Brunschwiler et al. review the role of two transcriptional regulators of major histocompatibility complex (MHC) class I and class II genes: the nucleotide-binding and oligomerization domain-like receptors (NLRs), NLR family CARD domain-containing protein 5 (NLRC5), and Class II Major Histocompatibility Complex Transactivator (CIITA). While NLRC5 regulates the expression of MHC class I molecules, CIITA regulates MHC class II. NLRC5 and CIITA also regulate some genes of the butyrophilin (BTN) family, which are involved in T cell activation, particularly in the activation of non-conventional Vγ9Vδ2 T cells by microbial metabolites. As some metabolites, such as phosphoantigens, are also released by transformed cells, Brunschwiler et al. propose that immunity evolved to distinguish self from “deregulated-self” i.e., metabolically abnormal cells. This interesting hypothesis comes along the “self-non self” and the “self-transformed self” hypotheses. It is not difficult to foresee great advances in this field.

Lastly, Sun et al. remind us that sepsis is a significant contributor to the global disease burden due to its high morbidity and mortality rates (nearly 11.0 million sepsis-related deaths just in 2017) and review the role of free heme in sepsis exacerbation. Sepsis is manifested as acute organ dysfunction and infection, which may progress to multiple organ failure and death; patients that survive sepsis may develop prolonged inflammation, immunosuppression, and organ damage, maintaining the risk of death. During the acute phase, patients may undergo hemolysis and systemic accumulation of hemoglobin which is then oxidized to methemoglobin (MtHb) by reactive oxygen and nitrogen species. Since MtHb is unstable in circulation, heme is then released as free heme, which participates in several forms of cell death (apoptosis, necroptosis, pyroptosis, ferroptosis, PANoptosis) as well as in inflammatory processes.

Based on free heme production and catabolic pathways, intracellular uptake, active metabolites and its role in cell death pathways, Sun et al. propose potential therapeutic targets for the treatment of sepsis, and summarize promising pharmaceutical candidates currently under investigation.

Wang et al. and Sun et al. papers in the present topics provide excellent examples of how a single metabolite may have a profound impact on health and disease.

## Challenges and the near future

With an estimated 10^23^ chemical reactions taking place in a living human body every second (8), it would be a rather difficult task to assess the real-time dynamics outcome from the administration of a physiologically relevant dose of any given metabolite; the task would still be very difficult if an isolated cell population is *in vitro* exposed to such a metabolite. Nevertheless, it is tempting to imagine that in both cases that intervention would lead to a metabolite-induced metabolic reprogramming. In the short run, an achievable task is to find links between final point metabolomics, cell function and single metabolite intervention *in vitro* as well as *in vivo*. It is likely that precise doses and metabolite combinations will progressively be used to boost or regulate immunity; this has to be supported by strong experimentation.
